# Sparse Optical Flow Implementation Using a Neural Network for Low-Resolution Thermal Aerial Imaging

**DOI:** 10.3390/jimaging8100279

**Published:** 2022-10-12

**Authors:** Tran Xuan Bach Nguyen, Javaan Chahl

**Affiliations:** 1 School of Engineering, University of South Australia, Mawson Lakes, SA 5095, Australia; 2 Aerospace Division, Defence Science and Technology Group, Edinburgh, SA 5111, Australia

**Keywords:** optical flow, thermal imaging, LWIR, navigation, UAVs, deep learning

## Abstract

This study is inspired by the widely used algorithm for real-time optical flow, the sparse Lucas–Kanade, by applying a feature extractor to decrease the computational requirement of optical flow based neural networks from real-world thermal aerial imagery. Although deep-learning-based algorithms have achieved state-of-the-art accuracy and have outperformed most traditional techniques, most of them cannot be implemented on a small multi-rotor UAV due to size and weight constraints on the platform. This challenge comes from the high computational cost of these techniques, with implementations requiring an integrated graphics processing unit with a powerful on-board computer to run in real time, resulting in a larger payload and consequently shorter flight time. For navigation applications that only require a 2D optical flow vector, a dense flow field computed from a deep learning neural network contains redundant information. A feature extractor based on the Shi–Tomasi technique was used to extract only appropriate features from thermal images to compute optical flow. The state-of-the-art RAFT-s model was trained with a full image and with our proposed alternative input, showing a substantial increase in speed while maintain its accuracy in the presence of high thermal contrast where features could be detected.

## 1. Introduction

The ability of unmanned aerial vehicles (UAVs) to navigate autonomously in unknown environments is vital for their further integration into human society. Currently, UAVs rely almost entirely on global navigation satellite systems (GNSS) for navigation applications. Nevertheless, GNSS are known to be unreliable in urban areas in urban canyons, or under forest canopies and are not available underground. Furthermore, GNSS systems do not provide any sensing capacity that might allow avoidance of unknown obstacles in the environment, thus making the solution less reliable in dynamic scenes.

Unlike other sensor-based systems, vision systems can provide real-time information about objects present in the scene. Furthermore, vision systems do not rely on signals coming from satellites such as GNSS, thus making it more resilient to conventional jamming [1]. Many researchers have demonstrated the potential of vision system for UAVs with promising results [2,3,4,5,6]. However, there remain challenges of using vision-based systems on UAVs due to the challenges of fusing spatial and temporal information from the sensors into a coherent model, which can be as simple as motion blur from high angular rates and lower rate of ground movement at higher altitude due to perspective [7] or lack of texture in scenes resulting in no information. Furthermore, due to high degrees of freedom of the UAVs, variations in roll, pitch and yaw of an aircraft with strap down cameras will result in different viewing angles and rates of image motion of the same scene captured from the same location [8].

A navigation system that can be deployed onto small UAVs must be small in size and light in weight. Small UAVs have limited payload capacity, which makes it difficult to utilise more computationally demanding algorithms for better accuracy. Some researchers have tried to solve this problem by using a cloud-based computer processing system to process data transmitted from UAVs in real-time [9,10,11,12]. However, this type of solution has limited range and cannot operate very far from the ground control station. Furthermore, it also shares the same unreliability issues faced by sensor based systems where the connections are not always available. Hence, it is necessary to explore new navigation algorithms that are less expensive for UAVs.

Optical flow can be defined as the apparent motion of brightness patterns across two frames [13]. Optical flow is a computer vision technique that is often associated with insect inspired studies [2,14]. Flying insects are able to navigate in a dynamic environment with a tiny brain [3]. Furthermore, insects have been shown to rely on optical flow for takeoff and landing [2,15], obstacle avoidance [16], terrain following [17] and flight speed regulation [18]. In navigation, optical flow can be used actively as frontal obstacle avoidance and altitude control [6], or passively to collect current operating states of the aircraft such as pitch and roll [19,20], descent angles [21] and direction of travel or lateral drift [5,22,23].

Over some decades, optical flow algorithms have been dominated by spatiotemporal image processing techniques to compute optical flow. Some examples include the Horn and Shunck technique [13], the Farneback algorithm [24], the gradient based such as the Lucas–Kanade [25], correlation and block matching methods [26] and the image interpolation technique [27] to name a few.

With ease of access to more powerful graphic processing units (GPU), scientists have been experimenting with optical flow implementations based on deep learning concepts with great success. FlowNet [28] was the first model in the field but its efficacy was inferior to traditional techniques. FlowNet2 [29] was created by stacking multiple FlowNet layers, which vastly increased the efficacy and outperformed many traditional methods, but required much more memory, making this approach unsuited to current embedded systems on small drones. Later work focused on light-weight models by borrowing many popular concepts from traditional techniques. SpyNet [30] uses a coarse-to-fine approach while LiteFlow [31] relies on a brightness map to solve occlusion problem. PWC-Net [32] utilises stereo matching, feature extraction and cost volume resulting in high efficacy while having substantially smaller model size compared to FlowNet2. Most recently, the Recurrent All-Pairs Field Transform (RAFT) [33] and its lighter version, RAFT_s, was introduced that achieved state-of-the-art efficacy while also having one of the lowest memory requirements. The RAFT models were inspired by optimisation-based approaches from traditional optical flow techniques.

While research activity is significant, there is a substantial gap in the literature into night operation given that this period represents approximately half of the potential operating time of a system. There are many reasons for this, such as the historically high cost of thermal sensors, the difficulty to operate after dark due to regulatory restriction and challenges to launch and retrieval of small aircraft at night.

In this paper, we explore a simple but effective technique that further enhances the performance of the RAFT_s model in terms of how many frames can be processed per second with thermal imagery. This technique can potentially further decrease the computational requirement of deep learning based optical flow techniques, which makes it suitable for aerial navigation applications where a dense flow field is often unnecessary.

## 2. Related Work

Thermal imaging has various advantages over visual light spectrum in some applications, not only to aid navigation in challenging lighting scenarios but also to reveal information that is invisible to the naked eye. The physics fundamentals of thermal sensors and their advantages and disadvantages are well documented in [1,34,35].

Beside being studied for autonomous navigation, thermal imaging has been used to monitor railway infrastructure [36], monitor crops [37,38,39], driver monitoring [40], face recognition [41], vital sign extraction [42] and for COVID-19 detection [43,44] just to name a few.

In the navigation domain, earlier work has demonstrated encouraging results when combining long-wave infrared (LWIR) with optical light wavelengths to detect hidden features in dark scene. Maddern et al. [45] relied on LWIR thermal sensor to enhance the tested system over long periods of time by compensating the adverse effects from solar glare on RGB images. Brunner et al. [46] used thermal data to detect and reject poor features such as dust and reflective surfaces that are visible in the visual spectrum. Mouats et al. [47] proposed a multispectral stereo odometry system for unmanned systems and later developed a purely thermal stereo odometer for UAVs [48]. In the odometer, a pair of thermal sensors were located in front of the UAV to capture data, and were later used for feature matching and thermal 3D reconstruction. The results demonstrated that a thermal odometry system could produce comparable outcomes to a standard visible spectrum system.

While these results contributed to the field, they will struggle in some scenes when significantly hotter or cooler objects enter and leave the scene, due to lack of a high dynamic range calibrated radiometric sensors. The automatic gain control (AGC) system is provided with thermal sensors to compensate for drastic changes in the overall pixel intensity of the thermal image. However, they are designed to support the display of information to humans with limited ability to resolve intensity variations in a scene. The gain control causes substantial changes in scene contrast to accommodate the range of temperatures of objects in the scene.

Modern radiometric thermal sensors produce high bit depth images to represent thermal emissivity. A radiometric sensor is calibrated against a standard black body by the manufacturer, greatly improving the consistency and accuracy of thermal data [49]. Furthermore, these sensors are small, light and have low power consumption, which makes them suit the weight and size constraints of UAVs.

Using these radiometric sensors, Khattak et al. [50] proposed a multi-spectral fusion approach combining optical light and LWIR sensors to allow small UAVs to navigate in a dark tunnel without the presence of GNSS. The drone carried a short range illumination source for the optical sensor while the thermal sensor provided thermal data at longer range. They later developed a thermal-inertial system [51] which was capable of results comparable to other state-of-the-art visual methods used in mines underground.

Khattak et al. [52] also proposed a low-cost solution using thermal fiducial markers to help UAVs navigate reliably in a laboratory environment. The team [53] later developed a purely thermal-inertial system that utilised full 14 bit radiometric data. The study demonstrated that by using direct pre-AGC 14 bit thermal data, they could not only overcome the troublesome AGC process, but also increased the resilience and consistency of signals against loss of features over time.

Most recently, they [54] proposed fusion of thermal and visual inertial with a LIDAR (light detection and ranging) sensor to improve reliability for pose estimation. The team in [55,56,57] attempted to construct a 3D map of the environment with thermal sensor. They used a combination of range camera and thermal camera to collect real-time indoor data, to which 3D point clouds are matched via a RANSAC-based EPnP scheme. However, LIDAR and SLAM (simultaneous localisation and mapping) techniques are generally computationally expensive to run, making it undesirable for small UAVs. In an attempt to solve this issue, Lu et al. [58] presented an unsupervised deep learning network that can construct a 3D environment from thermal images for low light conditions, as an alternative technique to LIDAR sensor. The results showed this proposed technique is capable of providing a good 3D map in tested sequences, but those sequences are relatively simple.

Further, also utilising direct 14 bit thermal data, Shin and Kim [59] proposed a direct thermal-infrared SLAM algorithm to measure up to six degrees of freedom of the UAV. The results showed that the 14 bit data increased the robustness of the system in adverse lighting conditions.

The rest of this paper is organised into nine sections. Section 3 outlines our previous works and motivations for this study. Section 4 introduces the thermal sensor and revisits our technique to solve the brightness constancy problem associated with optical flow from thermal imaging. Section 5 outlines the difference between the spare and dense optical flow technique for aerial applications and the feature extraction algorithm used in this study. Section 6 presents our collected dataset and our method to generate ground truth from real-world data. Section 7 presents the neural network that was used in this study, the two RGB datasets, how the neural network was trained and the evaluation methodology. Section 8 and Section 9 report and analyse results from the experiment. Section 10 outlines lessons learnt and possible future research directions.

## 3. Motivations and Contribution

This study continues our work [60,61,62] to explore aerial applications of optical flow from low-resolution thermal images, “Thermal Flow”. Thermal Flow was implemented as a downward-looking system, mounted beneath the UAV, that output 2D optical flow vectors in X and Y displacement to track the movement of the platform. Thermal Flow was designed to mimic the output of the very efficient and popular PX4Flow [20] system, which can be integrated easily into available autopilot systems, such as the PixHawk [61].

RADAR systems such as circular scanning millimetre-wave [63,64,65,66] and LIDAR [67,68,69,70] have been implemented on UAVs, both of which are high-performance range sensors that emit electromagnetic energy at different frequencies. Emissions require substantial power from the platform, and in large systems, potentially levels of energy that are dangerous to humans and might interfere with other sensors or airspace users. Active sensors usually include effective range as one of their primary performance metrics, limited by power emitted and sensitivity to signal received. Passive sensing using computer vision techniques generally need platform motion or binocular separation and require onboard processing with sophisticated algorithms. There are advantages as well, including that range is limited only by platform motion (or platform binocular separation) and reflectance or illumination of the target, not by emitted radiation levels. All active sensors run the risk of having their emissions being detected in contested environments, indicating the presence of the UAV and potentially the nature of its activities to an adversary.

Our previous work relied on traditional optical flow techniques. On the other hand, deep-learning-based optical flow networks outperform traditional techniques in various synthetic datasets in several key benchmark criteria [71]. However, these techniques are very expensive to run and require an integrated GPU system to run in real time, which is not always suitable for aerial applications on small UAVs. Hence, in this study, we want to explore a new technique to further reduce the computational requirements of deep learning optical flow models.

The state-of-the-art RAFT_s model [33] was chosen due to it achieving very high accuracy with small memory footprint and fewest parameters. Nevertheless, the model is still computationally expensive compared to the very popular sparse Lucas–Kanade. One of the reasons is that the network was designed to produce a dense optical field, which is not necessary in this application of Thermal Flow, which only requires a single reliable 2D vector. Instead, a sparse technique is preferred over the dense technique due to its much less computational cost, so the onboard computer system can be smaller to satisfy physical constraints of UAVs.

To achieve this, we are inspired by the use of the Shi–Tomasi [72] algorithm by the Lucas–Kanade algorithm [25]. The features then were combined into a new image, with smaller size, to be used as an alternative input to the network. Since the input is smaller, this technique can potentially decrease the computational requirement for the task while maintaining accuracy. This study aims to bring a deep-learning-based optical flow network that outperforms traditional techniques onto UAVs by reducing the computational requirement.

## 4. Optical Flow with Thermal Imaging

This section outlines the thermal sensor used to collect the data in this study. This section also presents the problems with optical flow estimation when re-scaling to an 8 bit image format from 14 bit raw data, and revisits the technique to improve this process.

### 4.1. Thermal Sensor

All of the images used in this study were captured by the radiometric FLIR Lepton3 [73]. The Lepton3 is a long-wave infrared sensor, and was calibrated in manufacturing against a standard black body [49]. The sensor can output 160 × 120 pixels at 8.7 Hz. The sensor has a 56° field of view and has been shown to be adequate for airborne applications with small angular movements without further need for re-calibration [60].

### 4.2. Automatic Gain Control

Most thermal sensors, such as the FLIR Lepton 3 that was used to collect our data, output radiometric data in 14 bit format. On the other hand, the RAFT_s network, and available computer vision libraries, such as OpenCV [74], are designed to process 8 bit images. This is largely due to modern standard displays being designed to match the intensity discrimination of human observers [1]. The 14 bit raw data from the sensor must be converted into 8 bit to display on the screen or to work with available computer software.

**Figure 1:**
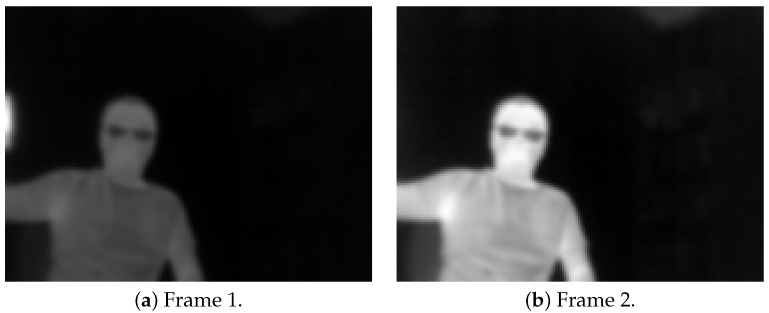
AGC changes the contrast in the images when a hot cup exits a scene: 1–2.

Thermal sensors such as the FLIR Lepton include an automatic gain control (AGC), which improves the contrast of the image when converting to 8 bit when there is a dramatic change in the temperature range present in an image. Figure 1 displays one example of a hot cup moving out of the screen, which shows a dramatic change in image contrast between consecutive images. This contrast change is likely to cause problem for many image matching algorithms. This change between frames violates the main assumption of optical flow equation, which is brightness constancy [13].

There were attempts to solve this problem. One approach [48] has been to greatly reduce the AGC response time so that image contrast does not change rapidly. Nevertheless, this technique only reduces the problem and does not solve it completely. Another group in [75] proposed an approach to manually set the range of the AGC. However, this approach requires prior information about the scene, which makes it less adaptable to unknown environments.

**Figure 2:**
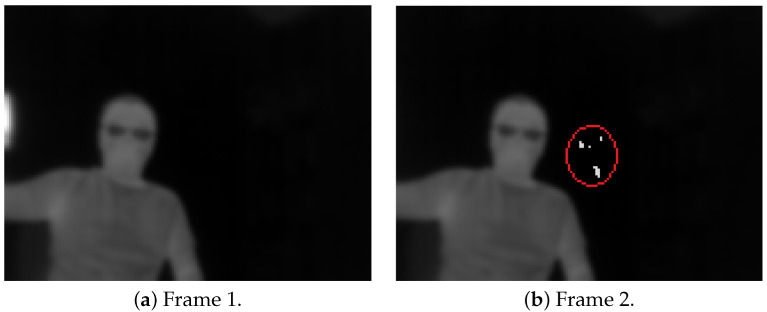
A pair from Figure 1 with our technique with small artefacts circled in red.

We revisit our conversion technique in [61] to re-scale two consecutive 14 bit images to 8 bit from the maximum and minimum pixel intensities found across both images. Figure 2 shows our technique. The technique, however, introduces some negligible artefacts.

Figure 2 shows the output of the sample images from Figure 1 with our technique. The contrast of the pair of images is maintained that can satisfy the optical flow requirement of brightness constancy.

## 5. Sparse and Dense Optical Flow Technique in UAV Navigation

A broad distinction can be made between dense and sparse optical flow techniques. Dense optical flow techniques are designed to compute an optical flow vector for every pixel within the image. On the other hand, sparse techniques only output optical flow for selected parts of the image. As a result, sparse techniques will typically require less computing resources than the dense techniques [76].

Thermal Flow is designed to mimic the output of the PX4Flow device, which is a 2D vector, flow_x and flow_y, which indicates the movement of the aircraft in X and Y displacements. The Thermal Flow system is intended to be mounted underneath the aircraft looking straight down possibly to augment navigation, which leads to a relatively simple optical flow field, compared to looking at shallower angles that might include the horizon. A dense optical flow field is not desirable in this application due to the high computational cost, which limits its use for small UAVs. The sparse technique on the other hand, has been shown to achieve sufficient accuracy for navigation applications in real life in various studies [2,5]. Therefore, it is possible that the sparse technique can be applied to greatly reduce the size of the data feeding into the neural network to reduce processing time while maintaining the accuracy.

### Feature Extraction

There are two primary feature extraction strategies: traditional corner-detection-algorithm-based and deep-learning-based frameworks. Deep-learning-based techniques include the direct visual odometry (VO) framework [77,78,79] and the 3D mapping mapping model [80,81], etc. Traditional techniques are based on grayscale changes in the images such as the Harris technique [82], and its improved version, the Shi–Tomasi algorithm [72]. In general, CNN-based algorithms can take three channel RGB images as input while the traditional techniques require the images to be converted to grayscale single channel format. Generally, the deep-learning-based algorithm have performed better than conventional techniques in challenging sequences but are also much more computationally expensive to run.

Since thermal images are in single channel grayscale format, and this study focuses on improving speed, the Shi–Tomasi technique was selected as the feature extractor in this study since it works well in practice and is much cheaper to run.

Consider a sub-window in the image located at position (x,y) and the pixel intensity at this location is I(x,y). When the sub-window shifts to a new position with displacement (u,v), the pixel intensity at this position can be expressed as I(x+u,y+v). The difference in pixel intensities of the window shift can be expressed as:

(1) δ=I(x+u,y+v)−I(x,y)

For good features in the thermal image, the difference is normally high. Let w(x,y) be the weights of pixels over a window; we differentiate Equation (1) with respect to X and Y axes. The weighted sum multiplied by the intensity difference for all pixels in a window, E(u,v) can be defined as:

(2) $$E\left(u,v\right)=\sum_{x,y}^{}*w\left(x,y\right)*\delta^{2}$$

Applying Taylor series expansion to the first order, to the shift intensity, we have:

(3) $$I\left(x+u,y+v\right)\approx I\left(x,y\right)+\frac{\partial I(x,y)}{\partial x}u+\frac{\partial I(x,y)}{\partial y}v$$

Let:

(4) $$I_{x}=\frac{\partial I(x,y)}{\partial x}$$

and

(5) $$I_{y}=\frac{\partial I(x,y)}{\partial y}$$

Equation (2) becomes:

(6) $$E\left(u,v\right)=\sum_{x,y}^{}*w\left(x,y\right)*{\left(I_{x}u+I_{y}v\right)}^{2}=\sum_{x,y}^{}*w\left(x,y\right)*\left[{\left(I_{x}u\right)}^{2}+{\left(I_{y}v\right)}^{2}+2I_{x}I_{y}uv\right]$$

Rewriting Equation (6) in Matrix notation gives us:

(7) $$E\left(u,v\right)\approx \left(u,v\right)M\left(\frac{x}{y}\right)$$

Hence:

(8) $$M=E\left(u,v\right)\left(\begin{array}{cc} \sum_{x,y}^{}I_{x}^{2} & \sum_{x,y}^{}I_{x}I_{y}\\ \sum_{x,y}^{}I_{x}I_{y} & \sum_{x,y}^{}I_{y}^{2} \end{array}\right)$$

The score for each window *R* can be found using the eigenvalues of the matrix, which can be expressed as:

(9) $$R=det\left(M\right)-K{\left(trace\left(M\right)\right)}^{2}$$

where:

(10) $$det\left(M\right)=\lambda_{1}\lambda_{2}$$

and

(11) $$trace\left(M\right)=\lambda_{1}+\lambda_{2}$$

In the Shi–Tomasi technique, *R* then can be found by:

(12) $$R=min(\lambda_{1},\lambda_{2})$$

The *R* value represents a quality value of the correspondent corner, where a higher value indicates that the corner is a good distinct feature. We relied on the implementation of the technique in OpenCV. The parameter values are shown in Table 1.

**Figure 3:**
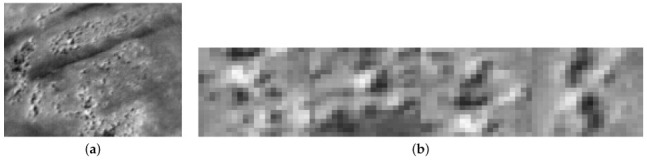
The original image (**a**) and the alternative input (**b**). The original size of (**b**) is 40 × 160 compared to (**a**) 160 × 120, which is one third of the number of pixels. (**a**) Sample thermal frame. (**b**) A new image constructed from extracted features from the original thermal frame with 40 × 40 as the cropping window and four features (shown at four times magnification).

The returned *R* value of the corner will be ranked, and the highest R value corners will be chosen first. After detecting good corners, a “cropping window” parameter will be applied to crop the surrounding pixels with the chosen corner at the middle, resulting in several sub-images. Then, these sub-images will be stitched together as an alternative image, as shown in Figure 3.

In some cases, the total number of features that can be found is less than the parameter value, such as only three found compared to four needed, then the algorithm will take three instead. If there are no features to be found, the software will set the flow vector value to zero.

## 6. Thermal Dataset Availability

To the best of our knowledge, there is no currently available 14 bit thermal dataset with optical flow ground truth. All the datasets that current networks use for training and validation are generated synthetic colour dataset with known ground truth. On the other hand, obtaining real optical flow ground truth from real-world data is extremely challenging [71] due to the high degrees of freedom of UAVs.

**Figure 4:**
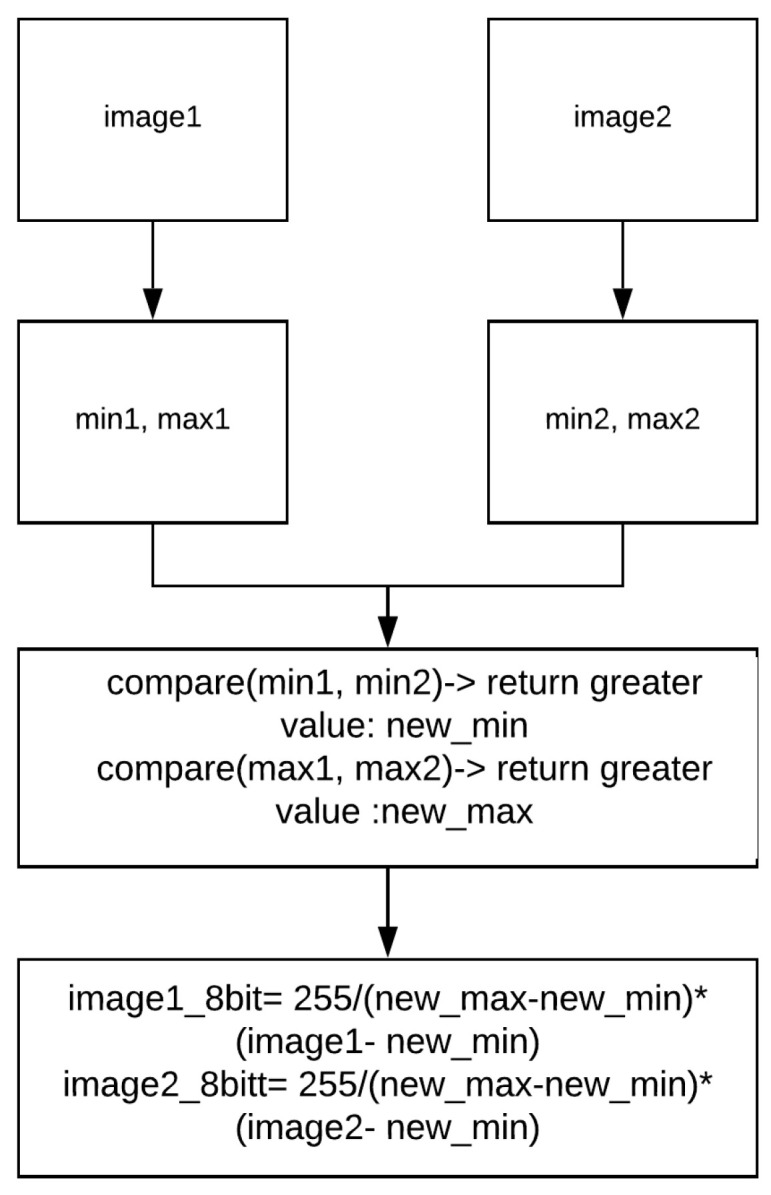
The 14 bit to 8 bit downsampling technique from [61].

To solve this problem, we generated ground truth from real-world thermal 14 bit data we had collected [60,62]. The 14 bit raw thermal data were downsampled to 8 bit with our technique as shown in Figure 4. After that, the traditional dense optical flow technique, Farneback [24], was used to generate ground truth from the images.

Although civilian drones are restricted to daylight hours under visual flight rules, military missions must be possible in all weather at all times of day and beyond visual line of sight. Night flights were conducted with approval in military airspace described in [60].

### 6.1. Dataset 1

**Figure 5:**
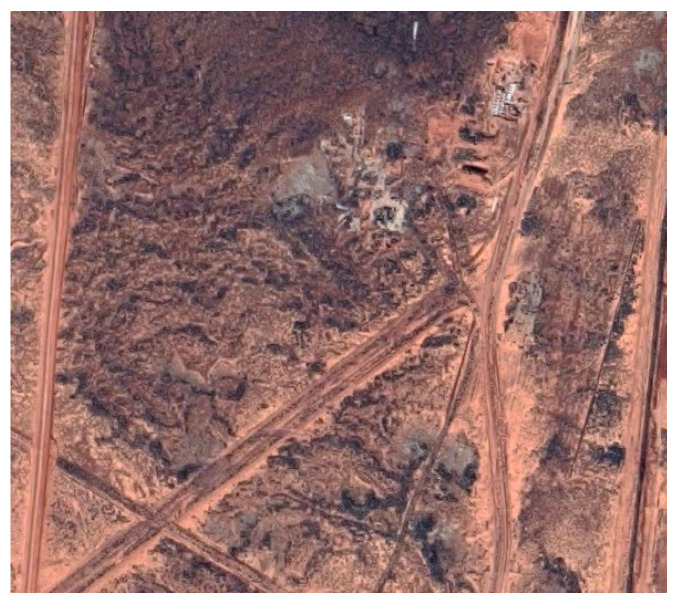
View of the field of Dataset 1.

Dataset 1 contained images from our work from [60], which includes 12,870 images captured above a flat arid field in northern South Australia. The data were captured in late summer, during clear and hot weather with the temperature at 34 C [83]. Figure 5 shows a colour image of the field.

**Figure 6:**
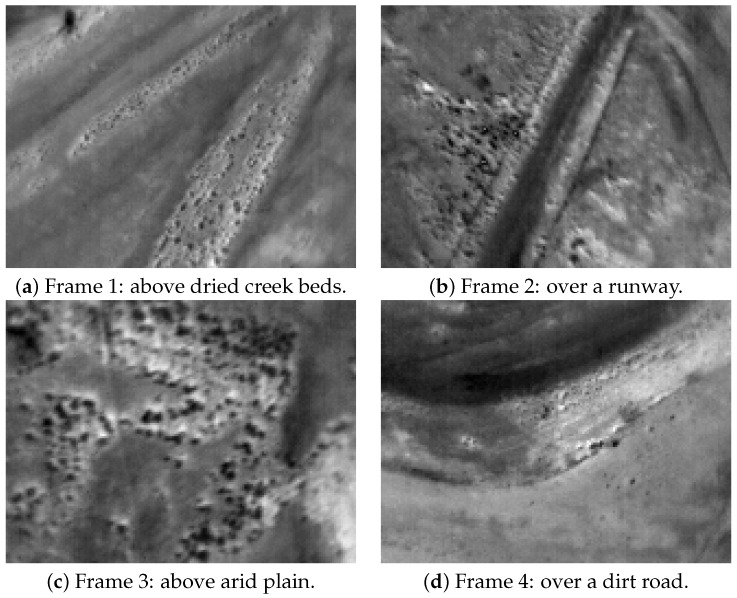
Some thermal frames of dataset 1, over some interesting features of the field.

Figure 6 shows some 8 bit thermal images of Dataset 1.

### 6.2. Dataset 2

**Figure 7:**
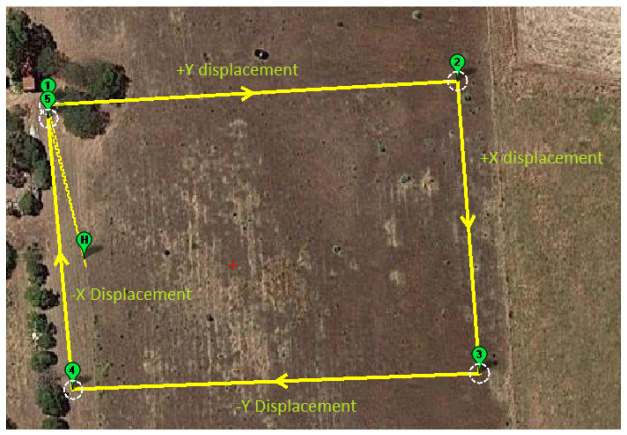
View of the environment of dataset 2 with its flight path in Mission Planner.

Dataset 2 contained a total of 2800 images from our work in [62], from an empty field on a hill in South Australia. The site provides a clear view of the sky and an empty ground with minimal artificial objects. Figure 7 shows the flight path in this experiment. The UAV took off at point H, flew to point (1)-(2)-(3)-(4)-(5) and then landed at (5).

**Figure 8:**
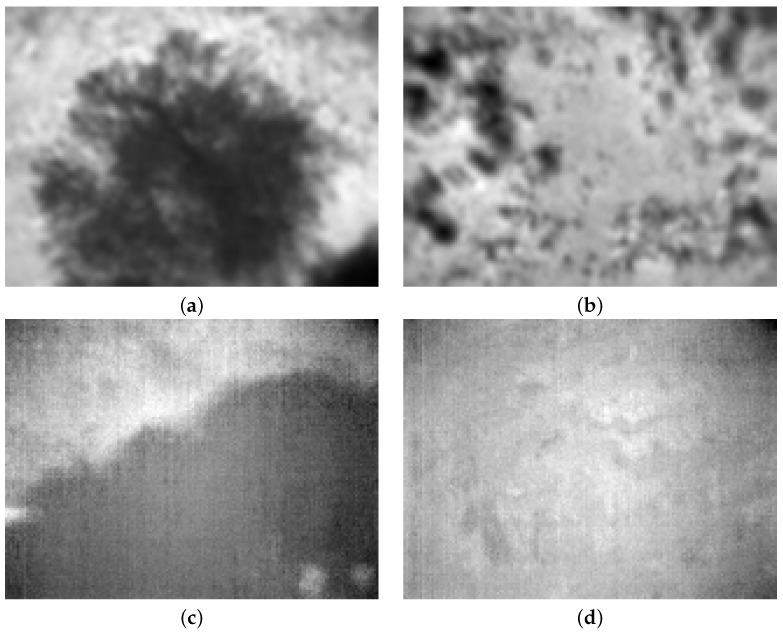
Some of images from dataset 2. Frame (1) and (2) shows the field during high-contrast conditions, and frame (3) and (4) shows thermal images at approximately at the same locations but under low-contrast conditions. (**a**) Frame 1: above a big tree during the high-contrast condition. (**b**) Frame 2: over an empty field during the high-contrast condition. (**c**) Frame 3: over a big tree during the low-contrast condition. (**d**) Frame 4: over an empty field during the low-contrast condition.

The data were captured at two different thermal contrast conditions: during a clear sunny day in late Autumn at 1600 h with high contrast in thermal data, and during a foggy rainy day during winter at 0900 h, which yields low thermal contrast. Hence, dataset 2 contains two smaller subsets of the same field that can be used to evaluate the performance of thermal flow during high and low-contrast conditions. Figure 8 shows some of the thermal images of the site under both conditions.

### 6.3. Training and Validating Sets

Table 2 shows a summary of our datasets, including how each dataset will be used for training and evaluation, number of images of each set and the conditions of the scenario.

In total, 10,894 images were used to train the RAFT_s network and 4800 images were used to evaluate the data during different thermal contrast conditions.

### 6.4. Generated ground truth from Thermal Dataset

**Figure 9:**
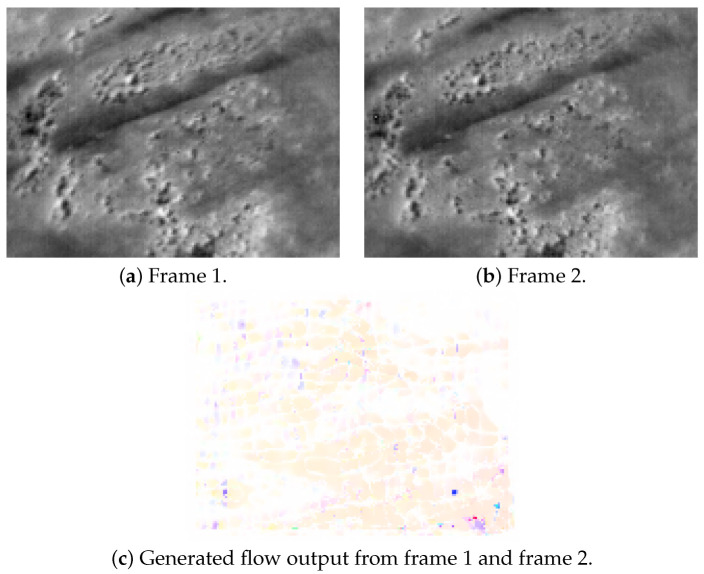
A sample sequence of thermal data and its generated ground truth from dense the optical flow Farneback algorithm in OpenCV.

Figure 9 shows a pair of images from dataset 1 and the dense optical flow ground truth generated by the Farneback technique implemented in OpenCV. To train the sparse network, the data from the dense network, including a pair of images, will be cropped at the location where coordinates of good corners were detected from Image1. Then, the respective sub-images will be aligned side by side from left to right to produce the training set for the sparse technique.

**Figure 10:**
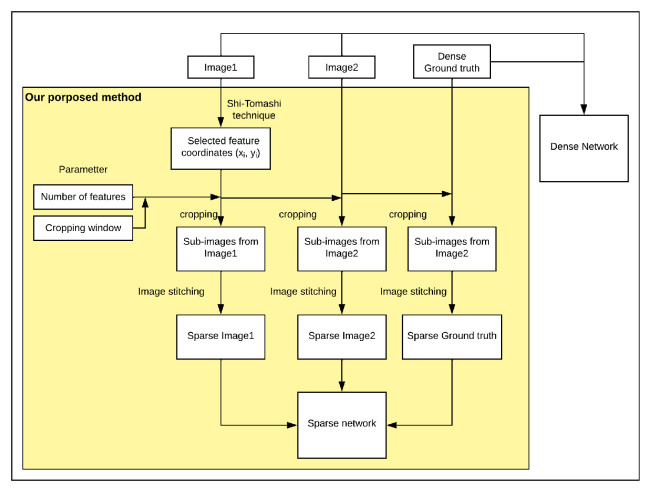
Flowchart shows our proposed technique. The dense network is trained with original images and ground truth. The big yellow block shows the process of our method to select good features with Shi–Tomasi technique, combine with predefined parameters to crop original frames and reconstruct new frames from those sub-images, which are the new inputs to the neural network.

**Figure 11:**
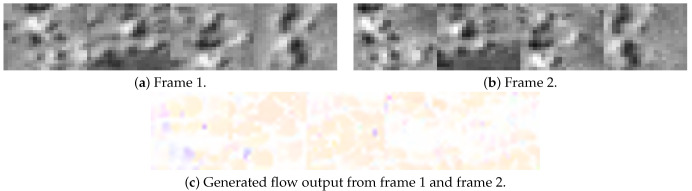
Sparse features used as input from images in Figure 9. The cropping window is 40 × 40 pixels and number of features is four. All images shown here were magnified for visual purposes.

The overall process is shown in Figure 10; Figure 11 shows the result from Figure 9.

## 7. The RAFT_s Model

The RAFT (recurrent all-pairs field transform) deep learning model consists of a composition of convolutional layers and recurrent layers in three main blocks: a feature context encoder, a convolution layer and a recurrent gated recurrent unit (GRU)-based layer.

The model extracts per pixel features and updates the flow field iteratively through a recurrent unit from the correlation volumes. In the feature extraction layer, two frames are taken as input where features are extracted separately similar to the FlowNetCorr model. The convolution layer consists of six residual layers with resolution halved on every second layer while the number of channels are increased. The model uses a single GRU with 3 × 3 filter in the GRU-based layer.

**Figure 12:**
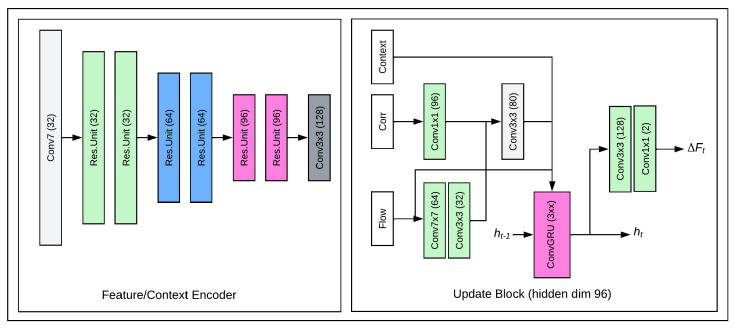
The model was used in this study [33].

Figure 12 shows the RAFT_s model used in this study.

### 7.1. RGB Optical Flow Dataset

We transferred learning with pre-trained weights from the MPI-Sintel final and flying chairs dataset. The flying chairs dataset was selected due to it containing a large number of images that represent 2D motion, while the MPI-Sintel final introduces more complex 3D motion under more challenging lighting conditions. While the dominant motion of the thermal dataset is 2D, there are still 3D motion effects in some scenes due to the large number of degrees of freedom of UAVs. Hence, introducing a dataset with some 3D motion is essential. The details of the two datasets are presented in the following sections.

#### 7.1.1. MPI-Sintel

**Figure 13:**
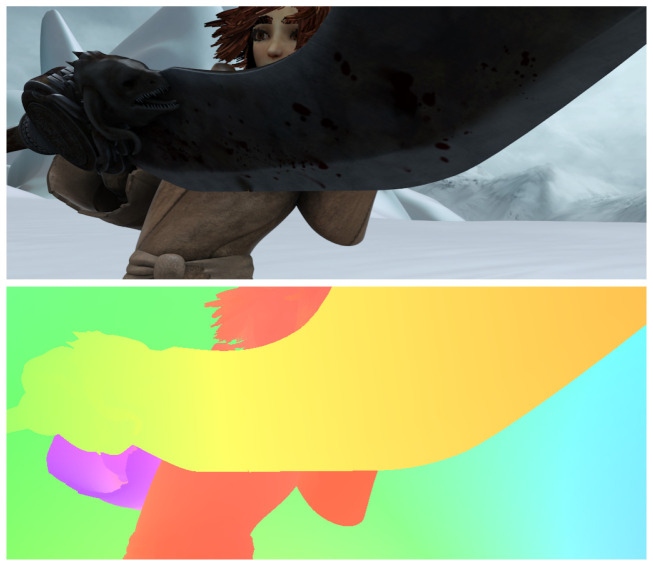
An example of an image (**up**) and its dense optical flow ground truth (**down**) from the MPI-Sintel final dataset.

Prior to 2015, MPI-Sintel [84] was the largest dataset for optical flow and disparity measurement. The frames within the dataset were extracted from open source 3D animated movies, so the MPI-Sintel is entirely synthetic. With high resolution at 1024 × 436 pixels, the frames included added effects from nature such as motion blur, fog or sun glare to make them more realistic. The training set consists of 1064 frames, which were divided into 23 training sequences. The evaluation set consists of 564 frames with 12 testing sequences. Dense optical flow ground truth is only available with the training set. The dataset provides three version: Albedo, clean and final. Albedo is the simplest set without any added effects, the clean version introduces small variation to the illumination between sequences and the final version adds more drastic effects. Researchers have been commonly using the clean and final versions over the Albedo. Figure 13 shows one example from the MPI-Sintel final dataset.

#### 7.1.2. Flying Chairs

**Figure 14:**
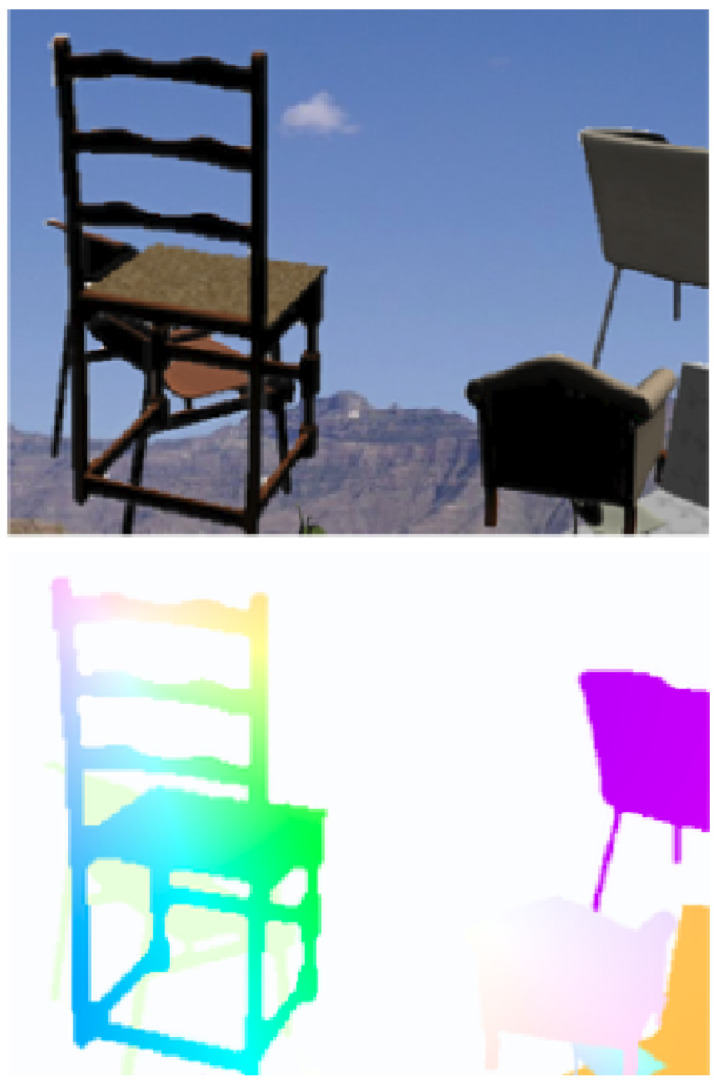
An example of an image (**up**) and its dense optical flow ground truth (**down**) from the flying chairs dataset.

The dataset were introduced along side the first deep-learning-based model, FlowNet [28]. It was designed specifically for training the deep learning network. The frames were constructed by placing 3D chair models above random backgrounds from Flickr. The dataset contains 22,872 frames with 22,232 for training and 640 for evaluation sets. The dataset is entirely synthetic and does not contain 3D motion, so it is limited to a single view only. Figure 14 shows one example from the dataset.

### 7.2. Train the Model

We transferred learning with pre-trained weights from the MPI-Sintel and flying chairs dataset, and our generated ground truth thermal data from the dataset as described in Section 6 and Section 6.4. The dense technique was trained with the ground truth from the whole image, as shown in Figure 9, and the sparse technique was trained with the cropped data, as shown in Figure 11.

For the technique that utilises the whole image, we labelled it “dense” to differentiate it from the model that uses the proposed technique, we call “sparse”.

The model was trained with the batch size of 10, with 160,000 steps, learning rate of 0.0001 and weight decay of 0.0001.

The network was trained on a computer with an Intel Core i7-7700 CPU, 64GB of RAM and Nvidia GTX 1080 Ti GPU. The operating system was Ubuntu 20.04, other programs including: pytorch version 1.6.0, torchvision 0.7.0, cudatoolkit 10.1, python 3.8 and OpenCV 4.5.5.

### 7.3. Evaluation Methodology

The dense and sparse models are evaluated on two criteria: Accuracy and speed. The speed is measured on how many frames per second (FPS) the network can process. The speed produced by the dense network is labelled as “dense FPS”, and the speed produced by our method is labelled as “sparse FPS”. The “difference” parameter is the percentage difference between the “sparse FPS” and the “dense FPS”. The “dense FPS” rate was 11 FPS from our experiment.

Accuracy is measured based on the normalised cross-correlation between the output signals from each model of the same image sequence. The normalised cross-correlation value is in the range [−1;1], with a value close to 1 indicating the two signals are the same, and vice versa.

There is a relationship between speed, cropping window and the number of features. A bigger cropping window and a larger number of features will likely return better accuracy but with lower speed. This relationship was investigated.

We learned that the cropping window at 40 × 40 pixels and the total of four features works reliably, and provide a balanced of speed and accuracy. Hence, these parameters are applied to the presented signals.

## 8. Result

This section presents the signals of the dense and sparse technique from the evaluation set. The parameter for the sparse technique is: “cropping window” at 40 × 40 pixels and four features.

### 8.1. Signals Accuracy

In this section, the overlay of dense and sparse signals over X and Y displacements and the normalised cross-correlation value are presented.

#### 8.1.1. Dataset 1

**Figure 15:**
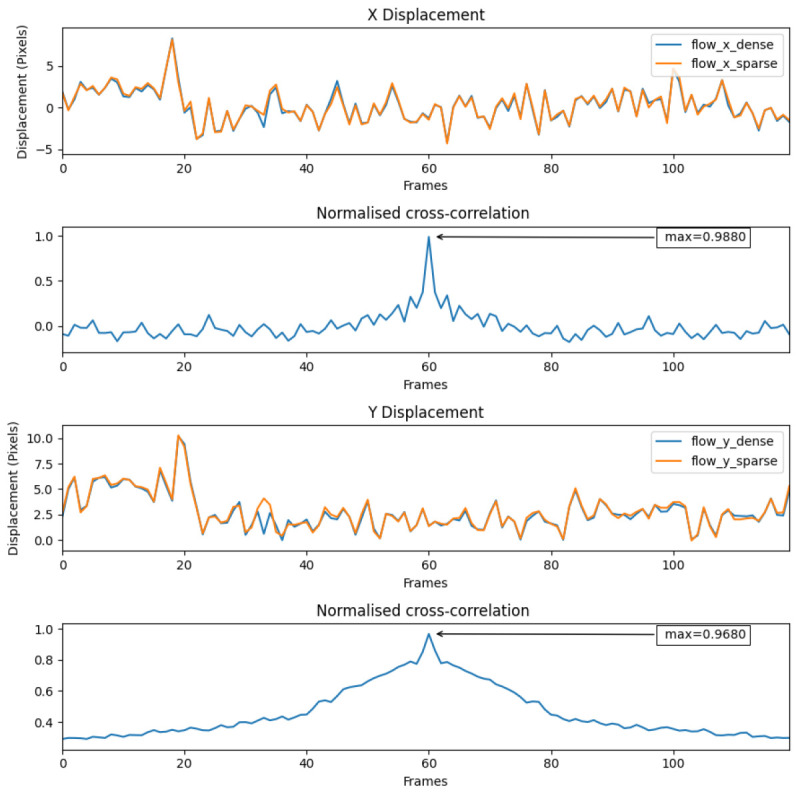
Dense and sparse technique signals on dataset 1.

Figure 15 shows the dense and sparse signals from the sequence of dataset 1. A very high normalised cross-correlation value of 0.988 in the X and 0.968 in the Y displacements indicating a strong relationship between the two signals, which means that the sparse technique are capable of maintaining accuracy with significantly less input data.

The average number of features found in this case matched the set value, which is four.

#### 8.1.2. Dataset 2 during High Thermal Contrast Conditions

**Figure 16:**
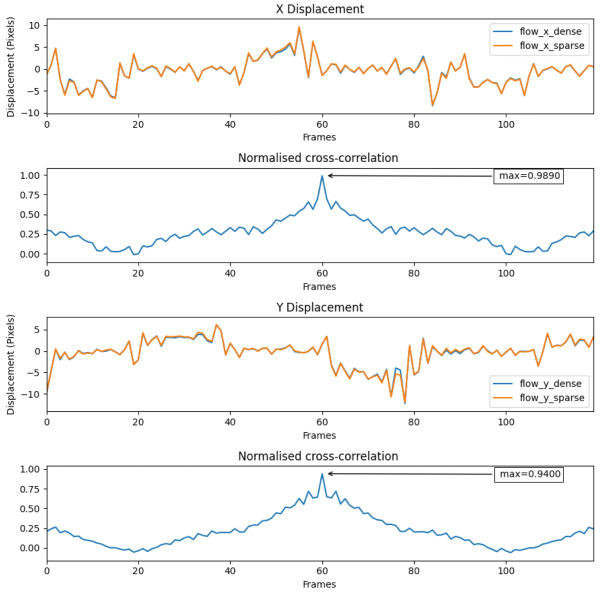
Dense and sparse technique signals on dataset 2 during high thermal contrast.

Figure 16 shows the dense and sparse signals from the sequence of dataset 2. A very high normalised cross-correlation value of 0.989 in X and 0.94 in Y displacements indicate a strong relationship between the two signals; thus, the sparse technique performs comparatively well in this scenario.

The average number of features found in this case also matches the set value, which is four.

#### 8.1.3. Dataset 2 during Cold-Soaked Low Thermal Conditions

**Figure 17:**
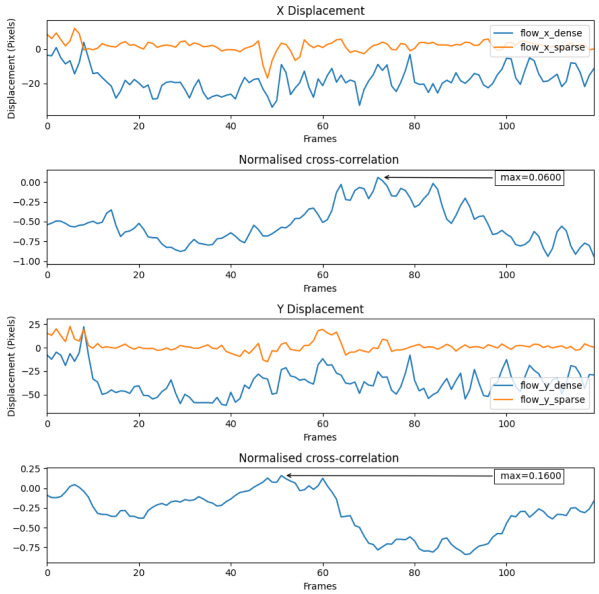
Dense and sparse technique signals on dataset 2 during low thermal contrast.

The number of features drops significantly; Figure 17 shows the dense and sparse signals from the sequence of dataset 2. A low normalised cross-correlation value of 0.06 in X and 0.16 in Y displacements indicating a very weak relationship between the two signals. Furthermore, most of the normalised correlation values in both X and Y displacements are negative, which indicates the sparse technique may not work well in low contrast environments. This result is consistent with our findings with other techniques in [61]. Since the Shi–Tomasi operator relies on contrasting features to detect good corners, this technique falls short in the the low contrast thermal condition.

Furthermore, since the Shi–Tomasi operator was not very effective, the number of features found was less than the set value of four. The average value of features found in this case was 1.83.

### 8.2. Effect of Cropping Window and Number of Features on Accuracy and Processing Time

This section outlines how changing the cropping window and number of features effects the accuracy and processing time of the model. These two separated cases are changing the cropping window while keeping the number of features constant and vice versa. Both cases were tested on dataset 1.

#### 8.2.1. Case 1: Constant Cropping Window

Table 3 shows the normalised cross-correlation values in X and Y displacements, processing time difference compared to the dense technique as a percentage. The results show that the accuracy in both X and Y displacement increase exponentially with the number of features in the image, until it reaches four features. After that, accuracy does not change significantly with higher numbers of features. The speed difference decreases steadily for each increase in number of features.

#### 8.2.2. Case 2: Constant Number of Features

Table 4 presents the results from changing the cropping window while maintaining four features. The sparse network achieves very low accuracy when the cropping window is less than 30 × 30. The accuracy increases significantly at 35 × 35 then peaks at 40 × 40. After that, the accuracy does not change significantly and even decreases at 55 × 55 pixels. The speed, meanwhile, steadily decreases with larger cropping window.

## 9. Discussion

The results show that the our proposed technique can be applied to a dense optical flow neural network for airborne applications with thermal imaging for faster processing time, while maintaining its accuracy. This technique can potentially reduce the computational demand of the network, which translates to a lighter payload and longer operating time for small UAVs.

The proposal relied on the Shi–Tomasi feature-based technique to detect good corners in the image, which does not work well in cold-soaked conditions. This is because these features rely on the difference in temperature between parts of the environment. We experimented with lower threshold values to detect low contrast features but the algorithm also picks up image noises at random sections within the image. The dataset in question was collected during winter, early in the morning of a rainy and foggy day. Hence, the temperature difference was low, which leads to lower thermal contrast. This is consistent with our findings in [61].

We learned that the sparse network can output comparable signals to the dense network in both X and Y displacements with four selected features combined with a cropping window at 40 × 40 pixels. The processing speed increased by 74.54% compared to the dense network. The sparse network does not work well with smaller value of these parameters, while larger values also do not increase the accuracy at the cost of higher computational cost.

## 10. Conclusions

This study showed that only using some good regions in a thermal image is enough to obtain a good 2D optical flow vector for certain airborne applications. Our study showed that this technique can decrease processing time by 74.54% while maintaining accuracy of the output.

However, due to the Shi–Tomasi technique relying on high contrast to detect good corners in thermal images, it does not work well under low contrast conditions. This issue potentially limits its use in some circumstances.

Future studies will look at other, versatile feature extraction techniques to solve the problem of thermal flow during cold-soaked low contrast condition.

## Figures and Tables

**Table 1:** Parameter settings for the Shi–Tomasi corner detection algorithm.

Feature Detection Setting	Maximum corners	1000
	Quality level	0.02
	Minimum distance	5
	Block size	5

**Table 2:** Characteristics of the collected dataset.

	Source	Training Set	Evaluation Set	Site Condition	Total Images
Dataset 1	[60]	Yes: 10,894	Yes: 2000	High contrast	12,894
Dataset 2	[62]	No	Yes: 2800	High and low contrast	2800
Total images		10,894	4800		15,694

**Table 3:** The effect of “number of features” on accuracy and processing time of the model.

No Features	Cropping	Cross-Correlation X	Cross-Correlation Y	Sparse FPS	Dense FPS	Difference
1	40 × 40	0.381	0.219	29.2	11	+165.45%
2	40 × 40	0.412	0.371	24.3	11	+120.9%
3	40 × 40	0.741	0.673	22.5	11	+104/54%
**4**	**40 × 40**	**0.988**	**0.969**	**19.2**	**11**	**+74.54%**
5	40 × 40	0.991	0.983	16.3	11	+48.18%
6	40 × 40	0.967	0.931	12.3	11	+11.81%
7	40 × 40	0.961	0.981	8	11	−27.27%

**Table 4:** The effect of cropping window on accuracy and processing time of the model.

No Features	Cropping	Cross-Correlation X	Cross-Correlation Y	Sparse FPS	Dense FPS	Difference
4	20 × 20	0.123	0.07	34.5	11	+213.64%
4	30 × 30	0.126	0.05	26.9	11	+144.54%
4	35 × 35	0.642	0.694	21.7	11	+97.27%
**4**	**40 × 40**	**0.988**	**0.969**	**19.2**	**11**	**+74.54%**
4	45 × 45	0.963	0.953	17.5	11	+55.09%
4	50 × 50	0.983	0.943	14.5	11	+31.81%
4	55 × 55	0.921	0.953	10.2	11	−7.27%

## Data Availability

Not applicable.

## Abbreviations

The following abbreviations are used in this manuscript:

LWIR	Long Wavelength Infrared
AGC	Automatic Gain Control
FFC	Flat Field Correction
$I^{2}A$	The Image Interpolation Algorithm
UAV	Unmanned Aerial Vehicle
LK	The Lucas–Kanade Algorithm
